# Molecular analysis demonstrates high prevalence of chloroquine resistance but no evidence of artemisinin resistance in *Plasmodium falciparum* in the Chittagong Hill Tracts of Bangladesh

**DOI:** 10.1186/s12936-017-1995-5

**Published:** 2017-08-15

**Authors:** Mohammad Shafiul Alam, Benedikt Ley, Maisha Khair Nima, Fatema Tuj Johora, Mohammad Enayet Hossain, Kamala Thriemer, Sarah Auburn, Jutta Marfurt, Ric N. Price, Wasif A. Khan

**Affiliations:** 1 Infectious Diseases Division, International Centre for Diarrheal Diseases Research, Bangladesh Mohakhali, Dhaka, 1212 Bangladesh; 2 Global and Tropical Health Division, Menzies School of Health Research and Charles Darwin University, Darwin, Australia; 3 Department of Zoology, University of Dhaka, Ramna, Dhaka, 1000 Bangladesh; 4 Centre for Tropical Medicine and Global Health, Nuffield Department of Clinical Medicine, University of Oxford, Oxford, UK

**Keywords:** *Plasmodium falciparum*, Antimalarial drug resistance, Molecular markers, Single nucleotide polymorphism, *pfcrt*, *pfmdr1*, Artemisinin, *K13* propeller, Bangladesh

## Abstract

**Background:**

Artemisinin resistance is present in the Greater Mekong region and poses a significant threat for current anti-malarial treatment guidelines in Bangladesh. The aim of this molecular study was to assess the current status of drug resistance in the Chittagong Hill Tracts of Bangladesh near the Myanmar border.

**Methods:**

Samples were obtained from patients enrolled into a Clinical Trial (NCT02389374) conducted in Alikadam, Bandarban between August 2014 and January 2015. *Plasmodium falciparum* infections were confirmed by PCR and all *P. falciparum* positive isolates genotyped for the *pfcrt* K76T and *pfmdr1* N86Y markers. The propeller region of the *kelch 13* (*k13)* gene was sequenced from isolates from patients with delayed parasite clearance.

**Results:**

In total, 130 *P. falciparum* isolates were available for analysis. The *pfcrt* mutation K76T, associated with chloroquine resistance was found in 81.5% (106/130) of cases and the *pfmdr1* mutation N86Y in 13.9% (18/130) cases. No single nucleotide polymorphisms were observed in the *k13* propeller region.

**Conclusion:**

This study provides molecular evidence for the ongoing presence of chloroquine resistant *P. falciparum* in Bangladesh, but no evidence of mutations in the *k13* propeller domain associated with artemisinin resistance. Monitoring for artemisinin susceptibility in Bangladesh is needed to ensure early detection and containment emerging anti-malarial resistance.

## Background

The World Health Organization (WHO) estimates that 3.2 billion people are at risk of malaria worldwide, with a total of 214 million malaria cases reported in 2015 [1]. Malaria is endemic in 13 districts of Bangladesh and approximately 80% of all cases are reported from the Chittagong Hill Tracts (CHT) on the south-eastern border with Myanmar [2]. More than 90% of all malaria cases are attributable to *Plasmodium falciparum*, with almost all other malaria cases are caused by *Plasmodium vivax* and mixed infections of both species [3]. Very few cases of *Plasmodium malariae* and *Plasmodium ovale* are reported from the CHT [4]. Rainy season (June–August) is considered as the peak transmission season of malaria in CHT [5]. A number of *Anopheles* spp. were found to be responsible for malaria transmission in CHT among those *Anopheles jeyporiensis, An. maculatus*, *Anopheles vagus* and *Anopheles nivipes* might be playing important roles [6–8].

Bangladesh adopted the artemisinin-based combination therapy (ACT) artemether–lumefantrine (AL) for the treatment of uncomplicated falciparum malaria in 2004, as a consequence of increasing chloroquine resistance in *P. falciparum* [9], but it took another 3 years to implement this policy in the rural areas of the country [10].

A number of drug resistance mechanisms have been reported in *P. falciparum* [11, 12]. Chloroquine (CQ) resistance has primarily been associated with mutations in the gene encoding the *P. falciparum* chloroquine resistance transporter gene (*pfcrt*) [13], with additional minor determinants associated with polymorphisms of the *P. falciparum* multidrug resistance 1 gene (*pfmdr1*) [14, 15]. *Pfmdr1* polymorphisms have also been associated with decreased susceptibility to amodiaquine (AQ), lumefantrine (LM) mefloquine (MQ) and artesunate (AS) [16, 17]. Copy number amplifications of the *pfmdr*1 gene have also been shown to reduce susceptibility to lumefantrine, as well as mefloquine [17, 18].

The first clinical reports of artemisinin resistance in *P. falciparum* were documented on the Thai–Cambodian border in 2003 and 2004 [19–22], with subsequent genomic studies identifying mutations in the propeller domain of the Kelch gene (*k13*) as the key molecular marker [23]. Isolates with the *k13* polymorphism exhibit increased survival rates in the presence of artemisinin in vitro and are associated with delayed parasite clearance times following ACT in vivo [23]. Other non-synonymous polymorphisms in *mdr*2 (multidrug resistance protein 2), *crt* (chloroquine resistance transporter), *fd* (ferredoxin) and *arps*10 (apicoplast ribosomal protein S10) genes may also be involved since artemisinin resistance [24].

Despite the withdrawal of routine CQ treatment more than 10 years ago, the prevalence of CQ resistance markers in *P. falciparum* remains high in Bangladesh. Recent studies report the presence of the CQ resistance-associated mutation K76T in the *pfcrt* gene in 82–100% of all clinical samples collected in the CHT [10, 25–28], and the N86Y mutation in the *pfmdr1* genes in 19–70% of clinical samples collected between 2002 and 2013 [10, 25, 27, 28]. However to date, there has been no evidence for reduced efficacy of ACT in Bangladesh. A study conducted on parasites collected between 2009 and 2013 identified a non-synonymous mutation (A578S) in the *k13* gene in a single isolate in an endemic area of the CHT [29], but the C580Y mutation linked to delayed parasite clearance in Cambodian isolates was not present [23, 29]. Subsequent analyses have shown that the A578S allele is not associated with clinical or in vitro resistance to artemisinin [30].

Reduced artemisinin efficacy has been reported from neighbouring countries. A study conducted in 2013 in Kayin State, in south-eastern Myanmar found 26% of patients treated with AL were still parasitaemic on day 3. However, the same study found no evidence of delayed parasite clearance at a second site on the western Myanmar border with Bangladesh [31]. Another study conducted between 2013 and 2014 reported mutations in the *K13* propeller region in 6.9 and 3.2% of samples collected in Rakhine and Chin State of Myanmar, both bordering Bangladesh, but no mutant isolates were detected in cross-border samples from the CHT [32]. A recent study from India confirmed the presence of F446I, A578S and K189T mutations in Mizoram and Tripura states which are close to the Bangladeshi border [33].

The objective of this study was to assess the present status of molecular variants suggestive of artemisinin resistance and ongoing CQ drug resistance in *P. falciparum* samples collected in the CHT.

## Methods

### Study area and population

Parasite isolates were collected from patients enrolled into a prospective, health care facility-based observational study to assess the efficacy of the current anti-malarial policy conducted in Alikadam, a sub-district of Bandarban district in the CHT. The study was conducted between September 2014 and January 2015 [34]. Ethical approval for this study was obtained from Ethical Review Committee of icddr,b (PR-14053) and the Human Research Ethics Committee of the Northern Territory Department of Health and Menzies School of Health Research, Australia (HREC2014-2228) as part of a clinical trial registered at clinicaltrials.gov Registration Number: NCT02389374.

### Sample storage and DNA extraction

Approximately 5 ml of whole blood were collected from participants enrolled in the clinical study; samples were preserved in EDTA tubes and stored at −20 °C for subsequent molecular analysis. DNA was extracted from 200 µl whole blood using the QiaAmp blood mini kit (QIAGEN, Inc., Germany) according to the manufacturer’s instructions. Extracted DNA of all samples was preserved at −20 °C until use.

### Species identification

*Plasmodium* species was confirmed by nested PCR following standard protocols [35] with minor modifications in the thermal cycles (35 cycles for Nest-1 and 40 cycles for Nest-2). Two genus specific primers were used to amplify 18ssrRNA gene and then species specific primers were used to amplify size specific portions of the ssrRNA sequence of Nest 1 that corresponds to *P. falciparum, P. vivax, P. malariae* and *P. ovale.*

### Genotyping of *pfcrt* and *pfmdr1*

The presence of the *pfcrt* (K76T) and *pfmdr1* (N86Y) mutations were assessed in all isolates using PCR–RFLP, following the protocol of Veiga et al. [36] with modifications to the extension temperature from 72 °C to 68 °C for *pfmdr1*.

### Sequencing of the *k13* propeller gene

The presence of *k13* mutations was assessed in a subset of 10 samples which exhibited the longest parasite clearance times recorded in the clinical study. The time interval between drug administration and first negative malaria slide was determined as parasite clearance time. [34]. The *k13* propeller gene region was amplified by nested PCR following the procedures described by Ariey et al. [23]. The nest-1 product was amplified with an expected product size of 849 bp and purified by ExoSAP-IT (Affymetrix). Amplified products were sequenced using the Sanger sequencing method on an ABI3500 Genetic Analyzer (Life Technologies). Both forward and reverse strands were sequenced to identify and confirm variants. The BioEdit Sequence Alignment Editor (ver. 7.0.9.0) was used to analyse the sequences. The sequences were aligned against the *P. falciparum* 3D7 strain (PF3D7_1343700, PlasmoDB Release 28) using ClustalO [37, 38].

All molecular analysis were performed at the Emerging Infections and Parasitology Laboratory of icddr,b. *k13* sequencing was performed at the Virology Laboratory of icddr,b.

### Genotyping to characterize recurrent infections

Two patients had recurrent *P. falciparum* parasitaemia after the follow up period of the primary study had ended [34], 32 and 36 days after the diagnosis of their initial infection. To assess whether the recurrent cases represented recrudescence or re-infection events, genotyping was conducted at the *msp1* (K1 of Block-2), *msp*2 (3D7 of central domain), and *glurp* loci following standard protocols [39, 40]. Taq polymerase, PCR buffer, dNTPs and restriction enzymes were sourced from New England BioLabs Inc, USA and a Bio-Rad C1000 thermal cycler was used for amplification. Nested amplicons and post-RFLP products were visualized under UV light on a Biorad gel documentation system after 1.5% agarose gel electrophoresis and ethidium bromide staining.

Following the WHO PCR-adjustment interpretation guidelines, recurrent infections were classified as homologous (recrudescent) if at least one allele was shared with the primary infection at each locus, and heterologous (re-infection) if no alleles were shared with the primary infection at 1 or more loci.

## Results

Of the 181 patients enrolled in the clinical, 180 samples (99.4%) were available for molecular analysis, of which 130 (72.2%) were confirmed as *P. falciparum* by PCR (119 *P. falciparum* mono-infection and 11 mixed infection with *P. vivax*). Two (1.6%) patients out of 122 were still parasitaemic on day 2 and were only seen again on day 6, by then they were aparasitaemic [34]. The median parasite clearance time was 22 h (range 14–40) [34], with ten isolates exhibiting clearance times greater than median parasite clearance times 22 h (range 24 to >48). Two patients had recurrent parasitaemia after the end of the study (day 32 and day 36 days), both of which were categorized as new infections by 3 loci genotyping.

### *Plasmodium falciparum* drug resistant markers

*pfcrt* and *pfmdr1* genotyping was successful in all of the 130 *P. falciparum* isolates. The *pfcrt* 76T mutation was present in 106 (81.5%) samples, with no sample indicating a mixed infection with both allelic variants. The *pfmdr1* 86Y mutation was present in 8 (6.15%) isolates, with an additional 10 (7.69%) isolates having a mixed infection with the 86Y and wild-type 86N allele. The 10 isolates from patients with prolonged parasite clearance times underwent sequencing of the *k13* gene. No synonymous and non-synonymous mutations were observed in *k13* in any of these isolates. All ten sequences were submitted to GenBank (Accession Numbers KY274188–KY274197).

## Discussion

The molecular analysis of isolates collected from patients with clinical malaria in the Chittagong Hill Tribes of Bangladesh highlights a high prevalence of *pfcrt* mutations, but reassuringly absence of the K13 mutations associated with artemisinin resistance. These findings concur with the corresponding clinical results published previously [34].

Although CQ was removed from the national treatment guidelines for *P. falciparum* malaria more than 10 years ago, the prevalence of CQ resistant *P. falciparum* strains remains high [10, 25–27, 41, 42]. In Malawi, Tanzania, Zambia and Grande Comore, CQ sensitive strains have become the predominant strains of *P. falciparum* 6–10 years after the withdrawal of CQ [43–48], but these reports have been in locations where chloroquine has been almost completely removed from the market, including the private sector. Similar observations of the reversion to chloroquine sensitivity have not been widely reported in Asian countries [27, 28, 32, 49–51]. In Bangladesh, CQ continues to be available in drug stores for self-treatment of malaria and for the treatment of *P. vivax*, and this drug use may have maintained CQ pressure on the local *P. falciparum* population, explaining the high prevalence of *pfcrt* 76T mutants in this study. The rate of 76T *pfcrt* mutations reported in this study population was comparable to rates reported from other areas of the CHT [10, 25–27].

None of the samples selected for sequencing of the *k13* gene had any single nucleotide polymorphisms (SNPs) in the propeller region indicative of reduced artemisinin sensitivity. The corresponding median parasite clearance times of 22 h (IQR 15.2–27.3 h) [34] and the lack of any treatment failure within 28 days of artemether lumefantrine suggest that the current malaria treatment guidelines for *P. falciparum* malaria remain highly effective. This result is in congruence with previously published data where almost all the patients were afebrile within 48 h [34].

Moderate prevalence of *pfmdr1* N86Y mutations have been reported earlier in studies conducted in Bangladesh [10, 27, 28, 52], however, the prevalence of this mutation in the current study was considerably lower. N86Y favours resistance to the aminoquinoline class of anti-malarial drugs including CQ [53]. Paradoxically, exposure to LUM may create selective pressure on the 86N wild type allele [54]. Thus, the results of our study reflects a selective force of ACT on 86N which may alter sensitivity to the drug.

If artemisinin resistance spreads westwards from the Mekong region, the CHT may be among the first areas affected of the Indian subcontinent. The recent influx of migrants from areas with a high prevalence of *P. falciparum* strains with reduced susceptibility to artemisinin, such as the bordering states of Rakhine and Chin in Myanmar [18], therefore, poses a significant threat to malaria control in this region. Furthermore Bangladesh continues to play a vital role in UN peacekeeping mission in sub-Saharan Africa, thereby providing a potential route for the spread of artemisinin resistance to the African continent [55]. Thus, constant monitoring of local anti-malarial drug efficacy in this region is vital to detect and prevent the spread of artemisinin resistance.

## Conclusion

The present study provides molecular evidence for the presence of CQ resistant *P. falciparum* in Bangladesh, but reassuringly no evidence of the key *k13* mutations associated with artemisinin resistance. Although the clinical and molecular data suggest that the current malaria treatment guidelines for *P. falciparum* remain highly effective, close monitoring for artemisinin and lumefantrine resistance will be critical in this malaria endemic area.

## Abbreviations

WHO: World Health Organization
*pfmdr1*: *Plasmodium falciparum* multidrug resistance 1
*pfcrt*: *Plasmodium falciparum* chloroquine resistance transporter
AL: artemether–lumefantrine
CQ: chloroquine
CQR: chloroquine resistant
ACT: artemisinin-based combination therapy
DNA: deoxyribonucleic acid
dNTP: nucleoside triphosphate
MQ: mefloquine
SNP: single-nucleotide polymorphism
PCR: polymerase chain reaction
RFLP: restriction fragment length polymorphism
